# Cytotoxicity Mechanisms of Eight Major Herbicide Active Ingredients in Comparison to Their Commercial Formulations

**DOI:** 10.3390/toxics10110711

**Published:** 2022-11-21

**Authors:** Scarlett Ferguson, Robin Mesnage, Michael N. Antoniou

**Affiliations:** 1 Gene Expression and Therapy Group, King’s College London, Faculty of Life Sciences & Medicine, Department of Medical and Molecular Genetics, Guy’s Hospital, London SE1 9RT, UK; 2 Buchinger Wilhelmi Clinic, Wilhelmi-Beck-Straße 27, 88662 Überlingen, Germany

**Keywords:** herbicides, glyphosate, Roundup, oxidative stress, genotoxicity, risk assessment

## Abstract

Commercial pesticide formulations contain co-formulants, which are generally considered as having no toxic effects in mammals. This study aims to compare the toxicity of 8 major herbicide active ingredients–namely glyphosate, dicamba, 2,4-D, fluroxypyr, quizalofop-p-ethyl, pendimethalin, propyzamide and metazachlor–with a typical commercial formulation of each active ingredient. Cytotoxicity and oxidative stress capability was assessed in human hepatoma HepG2 cells. Using an MTT assay, formulations of glyphosate (Roundup Probio), fluroxypyr (Hurler), quizalofop-p-ethyl (Targa Super) and dicamba (Hunter) were more toxic than the active ingredient alone. Metazachlor and its formulation Sultan had similar cytotoxicity profiles. Cytotoxicity profiles were comparable in immortalised human fibroblasts. Toxilight necrosis assays showed the formulation of metazachlor (Sultan50C) resulted in significant membrane disruption compared to the active ingredient. Generation of reactive oxygen species was detected for glyphosate, fluroxypyr, pendimethalin, quizalofop-p-ethyl, the formulation of 2,4-D (Anti-Liserons), and dicamba and its formulation Hunter. Further testing of quizalofop-p-ethyl and its formulation Targa Super in the ToxTracker assay system revealed that both products induced oxidative stress and an unfolded protein response. In conclusion, these results show that most herbicide formulations tested in this study are more toxic than their active ingredients in human tissue culture cell model systems. The results add to a growing body of evidence, which implies that commercial herbicide formulations and not just their active ingredients should be evaluated in regulatory risk assessment of pesticides.

## 1. Introduction

Pesticides include thousands of chemicals, which are sold to kill insects, nematodes, plants or microorganisms considered as a source of nuisance. Exposure to pesticides has been implicated in the development of human diseases causing cancer [1,2], Parkinson’s disease [3], Alzheimer’s disease [4], alterations in testicular function and reproductive performance [5], or changes in lipid or glucose metabolism which could be a source of metabolic diseases [5]. During sensitive periods of life such as pregnancy, pesticides can negatively impact foetal development and cause neurodevelopmental effects [6,7], or childhood cancers [8]. Despite this, regulatory tests have been relatively unsuccessful at predicting toxic effects of pesticides before commercial approval. An increasing number of studies are being published revealing health effects, which were not always detected in regulatory studies, for example such as the role of paraquat in Parkinson’s disease [3], the reproductive toxicity of the pyrethroid insecticides [9], non-Hodgkin lymphoma (NHL) caused by glyphosate-based herbicides (GBHs) [10], or neurological diseases caused by chlorpyrifos [11].

Pesticides are used worldwide as mixtures known as formulations. Commercial pesticide formulations not only contain the declared pesticidal active ingredient but also ‘co-formulants’. Co-formulants serve multiple purposes including increasing solubility of the active ingredient, acting as a surfactant to allow good adherence and coverage over the target organism and allowing penetration of the active ingredient through plant cell walls in the case of herbicides. The composition of co-formulants is frequently kept confidential by manufacturers as they deem them not to have innate pesticidal action and not toxic to non-target species, including humans. Crucially, for regulatory purposes, pesticide long-term toxicity studies in laboratory animals are only conducted with the active ingredients as declared by manufacturers and not with commercial formulations [12,13]. However, studies have shown that commercial formulations of pesticides are often more toxic than the active ingredient alone demonstrating that co-formulants can be toxic. In this regard there has been a major focus on studying the toxicity of GBHs, which are extensively used in agriculture, forestry, urban and domestic settings [14]. Compared to just glyphosate, GBHs can cause more oxidative stress [15,16], more DNA damage [17,18], and be more toxic [19], which changes the risk profile of this herbicide. GBHs have also been demonstrated to cause more reproductive [20,21,22] or hepatotoxic [23,24] effects as well as gut microbiome composition changes [25] compared to glyphosate alone. The greater toxicity of GBHs compared to glyphosate has been most graphically demonstrated in vivo using rat model systems. A multi-omics analysis of the liver of Sprague Dawley rats exposed for 2 years to an ultra-low dose of a Roundup GBH, revealed induction of marked oxidative stress as shown by an activation of glutathione-S-transferase and ascorbate free radical scavenger systems and associated non-alcoholic fatty liver disease [26]. In a follow-up investigation again in Sprague Dawley rats, demonstrated the representative formulation of glyphosate MON 52276 (European Union), and to a lesser extent glyphosate alone, increased hepatic steatosis and necrosis, providing further evidence that formulations of herbicides are more toxic than the active ingredients alone [24].

Although the toxicology of glyphosate and GBHs has become an area of intense research, the toxicity of other major herbicide active ingredients and their commercial formulations currently in use (Table 1) remains under-explored. This is a concern as there is evidence suggesting many frequently used herbicides can cause DNA damage [27,28,29,30,31] and oxidative stress [31,32,33]. An investigation of the genotoxicity potential of the herbicide 2,4-dichlorophenoxyacetic acid (2,4-D) and a commercial formulation in Chinese Hamster ovary (CHO) cells, found that this compound induced DNA-strand breaks in a dose-dependent manner [27]. Yin and colleagues found a significant dose-dependent induction of DNA damage in Chinese toad tadpoles (*Bufo gargarizans*) as a result of exposure to the herbicides acetochlor, butachlor, paraquat and methyl methanesulfonate [28]. The herbicide flurochloridone and its formulations Twin Pack Gold and Rainbow were found to give rise to a marked increase in the genetic damage index in HepG2 cells [29]. Mesotrione has been shown to induce DNA damage as well as an increase in reactive oxygen species (ROS), catalase (CAT) and superoxide dismutase (SOD) in common carp (*Cyprinus carpio*) [30]. Li and colleagues investigated the effects of the herbicide sulfentrazone on the earthworm (*Eisenia fetida*) and observed low DNA damage at day-21 along with a concentration-dependent increase in the production of ROS, SOD, CAT, guaiacol peroxidase and glutathione S-transferase (GST) [31]. In addition, Mesnage and colleagues employing the ToxTracker carcinogenicity assay system demonstrated that the herbicides 2,4-D and dicamba can induce an oxidative stress response [32].

Previous studies providing evidence of oxidative stress and DNA damage by certain herbicides, along with a lack of investigation into their toxicity, including genotoxicity, of those herbicide active ingredients and formulations listed in Table 1, highlight a major knowledge gap that needs to be addressed. This study aims to address this knowledge gap by investigating the toxicity of the UK’s 8 most used herbicide active ingredients compared to a representative corresponding commercial formulation. The focus of this investigation was on the mechanisms of toxicity, which can contribute to carcinogenicity. The International Agency for Research on Cancer (IARC) has established key characteristics of human carcinogens. These characteristics provide a framework for organising mechanistic data and assessing strengths and gaps in evidence, which can be used as a basis for toxicological screening in cell culture model systems. Based on the IARC guidelines we compared the effect of the selected herbicides and commercial formulations on cell viability, membrane degradation as a marker of necrosis, and induction of oxidative stress. Oxidative stress is of particular concern since if not strictly controlled can damage cells, proteins, and DNA, which can contribute to aging as well as leading to a range of diseases including cancer [33]. 

Our study provides the first information about the differential toxicity between major herbicides used in the UK and their active ingredients on selected markers, which can inform on the activation of carcinogenicity mechanisms by these pesticides.

## 2. Materials and Methods

### 2.1. Chemicals

The pesticide active principals were analytical grade and obtained from Merck Life Sciences (Watford, UK); glyphosate (N-(phosphonomethyl)glycine; glyphosate; CAS: 1071-83-6, catalog No: 45521), pendimethalin (CAS: 40487-42-1, catalog No: 36191), propyzamide (CAS: 23950-58-5, catalog No: 45645), metazachlor (CAS: 67129-08-2, catalog No: 36155), fluroxypyr (CAS: 69377-81-7, catalog No: 45758), 3, 6-dichloro-2-methoxybenzoic acid (dicamba; CAS: 1918-00-9, catalog No: 16826), quizalofop-P-ethyl (CAS: 100646-51-3, catalog No: 34074), and 2,4-Dichlorophenoxyacetic acid (2,4-D; CAS: 94-75-7, catalog No: 820451). The commercial formulations used were Roundup Probio (Monsanto UK Ltd., Cambridge, UK, 360 g/L glyphosate), Stomp aqua (BasF, 455 g/L pendimethalin), Kerb flo (DOW Agrosciences, 400 g/L propyzamide), Sultan 50C (Adama Agricultural Solutions LTD, Ashdode City, Israel, 500 g/L metazachlor), Hurler (Barclay Chemicals Manufacturing Ltd., Dublin, Ireland, 200 g/L fluroxypyr), Hunter (Agros Soluciones, 48 g/L dicamba), Targa Super (Nissan Chemical Europe SARL, St Didier Au Mont D Or, France, 50 g/L quizalofop-p-ethyl), Anti-liserons (BHS, 100 g/L 2,4-D). Stock solutions of the active ingredients were prepared in dimethyl sulfoxide (DMSO). Dilutions of the stock solutions that were administered to cells resulted in concentrations of DMSO below 0.5%. Serial dilutions of the active ingredients and formulations were prepared in appropriate tissue culture cell media.

### 2.2. Mammalian Cell Tissue Culture

The human hepatoma HepG2 cell line was obtained from the European Collection of Authenticated Cell Cultures (ECACC) and was used between passages 53 and 65. The human telomerase immortalised human fibroblast cell line was obtained from the American Type Culture Collection (ATCC) and used between passages 19 and 36. HepG2 cells were grown in high glucose, pyruvate Dulbecco’s Modified Eagle Medium (DMEM) (Thermo Fisher Scientific, Leicestershire, UK) supplemented with 10% foetal bovine serum (South American origin, Thermo Fisher Scientific), 2 mM L-glutamine (Thermo Fisher Scientific) and 100 units penicillin/mL and 100 µg/mL streptomycin (Thermo Fisher Scientific). The immortalised human fibroblasts were maintained in Dulbecco’s Modified Eagle Medium (DMEM) AQE (Thermo Fisher Scientific), 20% Medium 199 HEPES (Thermo Fisher Scientific), 10% foetal bovine serum and 100 μL hygromycin B (0.01 mg/mL, CAS 31282-04-9, TOKU-E, Belgium). Cells were cultured in 75 ${\mathrm{cm}}^{2}$ flasks (Corning, Tewksbury, MA, USA) under standard conditions of 37°C and 5% CO_2_ air atmosphere. For experimentation, cells were released using 0.5% trypsin-EDTA (Thermo Fisher Scientific) from stock flasks at no greater than 70% confluency and seeded into 96 well plates.

### 2.3. Cytotoxicity Assays

HepG2 and immortalised fibroblast cells were seeded at 50,000 cells/well in 100 μL of medium in clear 96 well plates. Following a 24 h incubation, cells were treated with the test substances diluted accordingly to the desired concentrations in tissue culture maintenance medium. After a further 24 h incubation, an MTT assay was performed to assess cell proliferation and thus cytotoxicity according to the manufacturer’s instructions. Cells were incubated for 2 h in MTT ([3-(4,5-dimethylthiazol-2-yl)-2,5-diphenyltetrazolium bromide] solution at 1 mg/mL in phosphate-buffered saline (PBS). The resulting formazan precipitate was then dissolved by addition of 100 µL DMSO and quantified spectrophotometrically at 560 nm using a GloMax Multi Microplate Multimode Reader (Promega, Madison, WI, USA). Cell viability was expressed as a percentage relative to the negative control, untreated cell samples consisting of cell culture medium only.

### 2.4. ToxiLight Cell Membrane Damage Assay

HepG2 hepatoma cells were seeded into black 96-well plates (Greiner Bio-One, Stonehouse, UK) at a density of 50,000 cells per well and incubated for 24 h. Following this, cells were treated with active ingredients or formulations at concentrations that produced a lethal concentration 50 (LC50) value of 50%, LC50/2 and (LC50/2)/2. If LC50 values could not be calculated, the highest three test concentrations from the MTT cell proliferation assays were used. Following incubation, the ToxiLight bioluminescent assay (Lonza, Slough, Berkshire, UK) was used according to the manufacturer’s instructions to assess cell membrane rupture and consequent necrosis. Briefly, a 50 μL aliquot of the adenylate kinase (AK) reagent was added to each well and after 5 min the plates were read using the GloMax Multi Microplate Multimode plate reader with excitation 485 nM and emission 520 nM settings. The background luminescence from wells with tissue culture medium alone was subtracted, and luminescence compared relative to the negative control, untreated cell samples consisting of cell culture medium only. Triton X100 (0.05%) was used as a cell membrane and necrosis-inducing positive control agent. 

### 2.5. Oxidative Stress

HepG2 cells were seeded at 10,000 cells/well in 80 μL medium in 96 well white-walled plates, incubated for 24 h and then treated with the test substances at the desired concentrations. The positive control, reactive oxygen species (ROS)-inducing agent treatment was 50 μM menadione, the negative control, untreated cell samples consisting of cell culture medium only. Immediately after treatment, 20 µL $H_{2}O_{2}$ substrate was added to each well. At 6 h post treatment, 100 µL ROS-Glo detection reagent (Promega, Southampton, UK) was added per well, as per the manufacturer’s instructions. The plates were then incubated at room temperature in the dark for 20 min and the luminescence read using the GloMax Multi Microplate Multimode plate reader at excitation 485 nM and emission 520 nM settings. 

### 2.6. ToxTracker Assay System

The ToxTracker assays [35] were undertaken as previously described [32]. First, wild-type mES cells (strain B4418) were exposed to 20 different concentrations of the test substances, with a maximum concentration of 10 mM to determine the cytotoxicity profile. Cytotoxicity was estimated by cell count after 24 h exposure by flow cytometry using a Guava EasyCyte Flow cytometer (Luminex‘s-Hertogenbosch, Hertogenbosch, The Netherlands) and was expressed as the percentage of viable cells compared to vehicle control exposed cells. From this dose response, 5 concentrations were selected for testing in the ToxTracker assay. The six independent mES reporter cell lines that constitute the ToxTracker system [28] were seeded in gelatin-coated 96-well cell tissue culture plates in 200 μL mES cell medium (50,000 cells per well). At 24 h after seeding, the medium was aspirated and fresh mES cell medium containing 10% foetal calf serum and the diluted chemicals was added to the cells. The response to the tested materials was evaluated at five concentrations in a 2-fold dilution series. Induced expression of the GFP reporter genes were determined after 24 h exposure by flow cytometry. Only GFP expression in intact single cells was determined. Mean GFP fluorescence was measured and used to calculate GFP reporter induction compared to a vehicle control treatment. Cytotoxicity was estimated by cell count after 24 h exposure and was expressed as percentage of intact, viable cells compared to vehicle exposed controls. For cytotoxicity assessment in the ToxTracker assay, the relative cell survival for the six different reporter cell lines was averaged as cytotoxicity levels were very similar. Metabolic activation was included in the ToxTracker assay by addition of S9 liver extract from aroclor1254-induced rats (Moltox, Boone, NC, USA). Cells were exposed for 24 h to five concentrations of the test compounds in the presence of 0.25% S9 extract and required co-factors (RegenSysA+B; Moltox). Positive reference treatments with cisplatin (DNA damage), diethyl maleate (oxidative stress), tunicamycin (unfolded protein response) and cyclophosphamide (metabolic activation of progenotoxins by S9) were included in all experiments. Solvent concentration was the same in all wells and never exceeded 1% for DMSO or 10% for water.

### 2.7. Statistical Analysis

The experiments were repeated a minimum of 3 times in different weeks (n = 9). Dose response on HepG2 and immortalised fibroblast cells was used to determine cytotoxicity thresholds using nonlinear dose-relationships. We determined the LC50, the concentration at which 50% of the cells are viable using best-fitted value of a nonlinear regression using sigmoid (5 parameter) equation in the GraphPad Prism 9 software package. 

Statistical differences of necrosis and oxidative stress were calculated using nonparametrc Mann–Whitney test also using GraphPad Prism 9 software.

Primary ToxTracker data were produced as flow cytometry (.fcs) files. Mean GFP expression and viable cell concentration after treatment were exported as text files (.csv) and imported into Microsoft excel for calculation of reporter gene induction and cytotoxicity. A positive response from ToxTracker is reported when a compound induces at least a 2-fold increase in GFP reporter gene expression in any of the six cell assay systems. Subsequent ToxTracker analysis was undertaken at concentrations that do not cause more than 75% cytotoxicity.

## 3. Results

**Figure 1:**
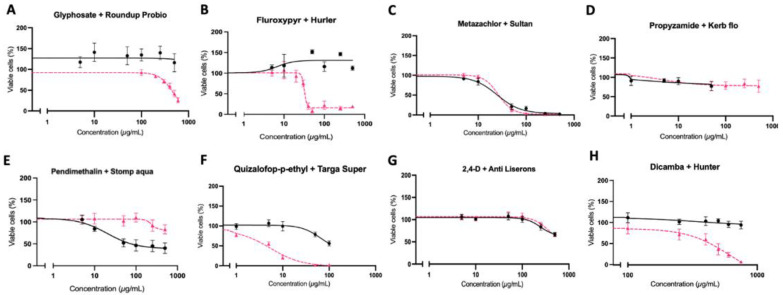
Differential cytotoxicity of herbicide active principles and their representative corresponding commercial formulations on human hepatocyte HepG2 cells using an MTT assay. Concentrations in µg/mL are dilutions of each active ingredient (black line) and formulation (pink line). The standard error of the mean (SEM) is shown in all instances (n = 15).

**Figure 2:**
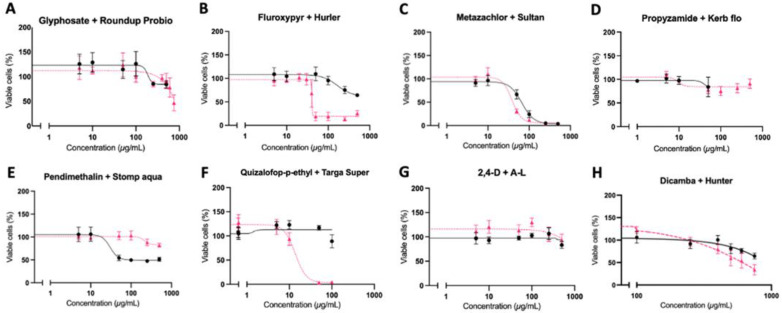
Differential cytotoxicity of herbicide active principles and their representative corresponding commercial formulations on human immortalised fibroblast cells using an MTT assay. Concentrations in µg/mL are dilutions of each active ingredient (black line) and formulation (pink line). The standard error of the mean (SEM) is shown in all instances (n = 15).

The toxicity of eight herbicide active ingredients and a corresponding typical commercial formulation on two human cell lines, HepG2 hepatocytes and immortalised fibroblasts, was studied using an MTT cell viability assay, which measures mitochondrial respiration via succinate dehydrogenase activity (Figure 1 and Figure 2). All pairs of pesticide active ingredients and formulations resulted in similar patterns of cytotoxicity in both cell lines. The formulations of glyphosate (Roundup Probio), fluroxypyr (Hurler), quizalofop-p-ethyl (Targa Super) and dicamba (Hunter) were more toxic than the corresponding active ingredient alone. LC50 values could be calculated for Roundup in HepG2 cells: 439.9 µg/mL (Figure 1A), for Hurler in HepG2 cells: 31.86 µg/mL (Figure 1B) and fibroblasts: 40.36 µg/mL (Figure 2B), Metazachlor in HepG2 cells: 24.88 µg/mL (Figure 1C) and fibroblasts: 66.28 µg/mL (Figure 2C), Sultan 50C in HepG2 cells: 26.35 µg/mL (Figure 1C) and fibroblasts: 38.26 µg/mL (Figure 2C), Targa Super in HepG2 cells: 5.39 µg/mL (Figure 1F) and fibroblasts: 13.73 µg/mL (Figure 2F) and Hunter in HepG2 cells: 620 µg/mL (Figure 2G). These results show the toxicity of the herbicide formulations is attributable to the co-formulants or a combination of the co-formulants and active ingredient rather than the active ingredient alone. Metazachlor was the only active ingredient tested, which displayed sufficient toxicity for an LC50 value to be calculated and was close to that of its formulation Sultan 50C in both cell lines. This suggests the toxicity of Sultan 50C is mainly due to its active ingredient metazachlor. 

**Figure 3:**
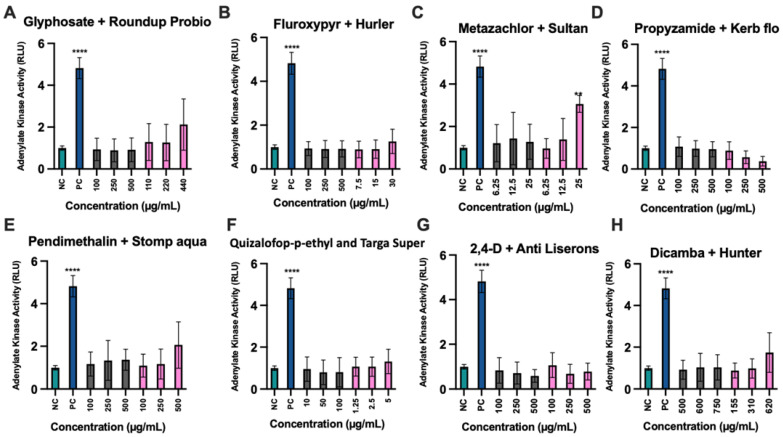
Measurement of membrane disruption cytotoxicity of herbicide active principles (grey columns) and their representative commercial formulations (pink columns) in human hepatocyte HepG2 cells. Untreated negative control cultures (NC), positive control cultures (PC) treated with 0.05% Triton X100 compared to each active ingredient (grey columns) and its respective commercial formulation (pink columns) 24 h after exposure. Adenylate kinase activity is expressed in relative units (RLU). Standard deviation (SD) is shown in all instances (n = 9). ** *p* < 0.001, **** *p* < 0.0001, in a Man-Whitney test which followed a one-way ANOVA.

We next undertook a ToxiLight cell membrane damage assay in an effort to gain a deeper understanding into the mechanisms of toxicity of active ingredients and their commercial formulations. The results obtained (Figure 3) showed that only the metazachlor-based formulation Sultan 50C at its LC50 concentration promoted significantly more membrane disruption compared to negative control samples (Figure 3C). This again showed that the commercial formulation Sultan 50C is more necrotic than just its active ingredient metazachlor.

**Figure 4:**
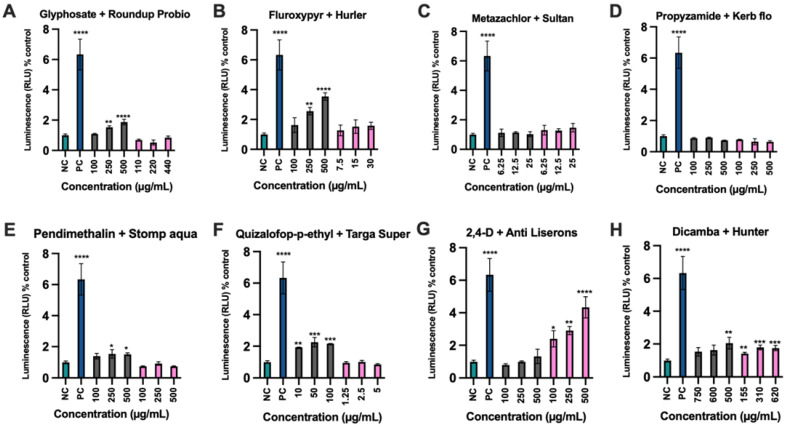
Herbicide-induced oxidative stress in human hepatocyte HepG2 cells. Levels of H_2_O_2_ production compared untreated negative control cultures (NC) and positive control (PC) 50 μM menadione treated cultures with each active ingredient (grey columns) and its respective commercial formulation (pink columns). Results are expressed as relative light units (RLU) from the luciferase reporter ROS-Glo $H_{2}O_{2}\;\mathrm{detection}\;$system. The assay was performed in triplicate and data are expressed as mean ± SD (standard deviation) of 3 independent replicates. * *p* < 0.05, ** *p* < 0.01, *** *p* < 0.001, **** *p* < 0.0001 in a Man-Whitney test which followed a one-way ANOVA.

The production of H_2_O_2_ was next investigated as an indicator of oxidative stress, to further explore mechanisms of toxicity. The positive control compound menadione resulted in a significant 6-fold increase in H_2_O_2_ production when compared to the negative control untreated samples (Figure 4). Glyphosate and fluroxypyr induced positive oxidative stress responses (*p* < 0.01) at concentrations of 250 µg/mL and 500 µg/mL (Figure 4A,B). All concentrations of quizalofop-p-ethyl 10 µg/mL (*p* < 0.01), 50 µg/mL and 100 µg/mL (*p* < 0.001) induced a significant positive oxidative stress response (Figure 4F). The formulation of 2,4-D (Anti-Liserons) induced a significant dose-dependent oxidative stress response at 100 µg/mL (*p* < 0.05), 250 µg/mL (*p* < 0.01), and 500 µg/mL (*p* < 0.0001) (Figure 4G). Significant oxidative stress responses were also seen with pendimethalin at 250 µg/mL and 500 µg/mL (*p* < 0.05) (Figure 4E). For dicamba a significant oxidative stress response was observed at 500 µg/mL (*p* < 0.01) and its formulation Hunter at 155 µg/mL (*p* < 0.01), 310 µg/mL and 620 µg/mL (*p* < 0.001) (Figure 4H).

**Figure 5:**
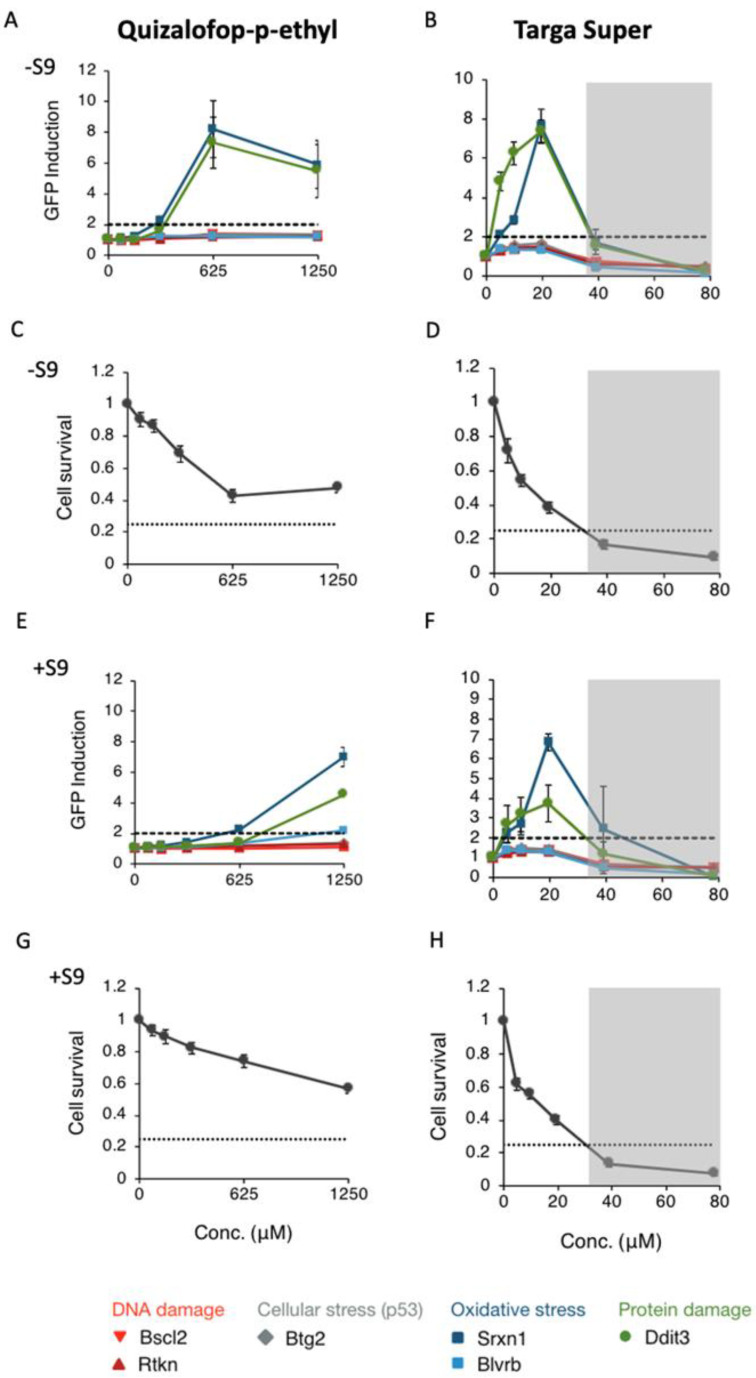
Activation of mechanisms known to be key characteristics of carcinogens by quizalofop-p-ethyl and Targa Super utilising the ToxTracker assay system. The six mES reporter cell lines that comprise the ToxTracker assay system were used to detect the activation of a DNA damage response (Bscl2 and Rtkn), oxidative stress (Srxn1 and Blvrb), protein damage and an unfolded protein response (Ddit3), and p53-mediated cellular stress (Btg2). Showing the induction of reporter gene expression in absence of S9 metabolising liver extract (**A**,**B**) and associated changes in cell survival (**C**,**D**). Induction of reporter gene expression in the presence of S9 liver extract (**E**,**F**) and cell survival (**G**,**H**). Grey box covers measurements with less than 25% cell survival and were not taken into consideration due to too high cytotoxicity. Dotted line is the threshold for a positive ToxTracker response.

Of the active ingredients evaluated, quizalofop-p-ethyl was particularly potent at inducing oxidative stress at all concentrations tested including at the lowest concentration (10 mg/mL) of any active ingredient (Figure 4F) suggesting a possible genotoxic capability. We therefore decided to investigate in greater detail the mechanisms of toxicity of this compound using the ToxTracker assay system (Figure 5). Initial cytotoxicity measurements of quizalofop-p-ethyl and its formulation Targa Super in normal mouse embryonic stem cells varied markedly, with Targa Super being notably more cytotoxic than its active ingredient alone in either the absence (Figure 5C,D) or presence (Figure 5E,F) of the metabolising S9 liver extract. Testing of quizalofop-p-ethyl and Targa Super in the ToxTracker assay system, showed that at the maximum tested concentration in the absence of a metabolising S9 liver extract, more than 50% cytotoxicity was observed for quizalofop-p-ethyl and Targa Super (Figure 5C,D). Neither of the two compounds activated the two reporter gene systems for genotoxicity nor the reporter for p53 gene activation sufficiently to pass the 2-fold threshold for a positive ToxTracker assay response (Figure 5A,B,E,F). However, significant activation of the oxidative stress reporter Srxn1-GFP was observed for both quizalofop-p-ethyl and Targa Super in the absence (Figure 5A,B) and presence (Figure 5E,F) of S9 metabolising liver extract. Activation of Blvrb-GFP was observed only in the presence of S9 extract (Figure 5E,F). In addition, the Ddit3-GFP reporter, which measures an unfolded protein response was activated upon exposure to quizalofop-p-ethyl and Targa Super in the absence (Figure 5A,B) and presence (Figure 5E,F) of S9 extract. 

## 4. Discussion

The aim of this study was to investigate the differential toxicity of active ingredients and representative commercial formulations of the eight most extensively used herbicides in the UK by comparing their effect on viability and biochemical parameters indicative of carcinogenesis in two human cell lines. Our results show that the commercial formulations of glyphosate (Roundup Probio), fluroxypyr (Hurler), quizalofop-p-ethyl (Targa Super) and dicamba (Hunter) brought about a much greater decrease in cell viability compared to the active ingredient alone (Figure 1 and Figure 2). The active herbicide ingredients glyphosate (Figure 4A), fluroxypyr (Figure 4B), pendimethalin (Figure 4E) and quizalofop-p-ethyl (Figure 4F) exhibited a significant oxidative stress response, which was not seen with their corresponding formulation. This may be due to higher concentrations of these active ingredients being applied to the cells as the three highest concentrations tested in the cytotoxicity assay (Figure 1) were used, as LC50 values for these substances could not be determined. Thus, a higher concentration of active ingredient alone was tested compared to the amount present in the respective formulation.

Most of the herbicide formulations evaluated in this study have not previously been tested for toxicity in human cell culture systems. Thus, our investigation is the first to compare the effects on cell viability of pendimethalin and any pendimethalin commercial formulation. Investigations of pendimethalin alone have shown a notable increase in oxidative damage and decrease in viability of human pancreas epithelial PANC-1 tumour cells [36], and a decreased viability of human umbilical vein endothelial cells [37]. This is consistent with the results we observed in both the HepG2 hepatocytes (Figure 1E) and immortalised fibroblasts (Figure 2E) where pendimethalin exposure resulted in a decrease in cell viability, and notably more so than its formulation. Additionally, we observed a significant oxidative stress response by pendimethalin but not its commercial formulation Stomp Aqua (Figure 4E). This is concordant with the cytotoxicity measures (Figure 1E and Figure 2E) and could offer an explanation as to the mechanism behind the greater decrease in cell viability seen from pendimethalin. According to their respective MSDS, declared co-formulants in Stomp Aqua (Table 1) are categorised H351 (suspected of causing cancer), Carc Cat 3 (Carcinogenic substances category and substances which cause concern for humans), owing to possible carcinogenic effects. Furthermore, Stomp Aqua has not had an assessment for carcinogenicity.

Numerous studies have compared pesticide active ingredients and respective commercial formulations in various cell lines. Gasnier and colleagues compared three glyphosate formulations namely Roundup Express, Bioforce and Grands Travaux plus and found all formulations tested resulted in a much larger decrease in cell viability than glyphosate alone in HepG2 hepatocytes [38]. Similar results were obtained in a study that compared glyphosate and the formulation Roundup GT+ in HepG2 hepatocytes, HEK293 and JEG3 cells where the formulation was found to be more toxic than glyphosate [13]. The results from these investigations are concordant with the outcome measures of the cell viability assays undertaken in this study, where we observed that the GBH Roundup Probio resulted in a larger decrease in cell viability than glyphosate alone in HepG2 hepatocytes (Figure 1A) and immortalised fibroblast (Figure 2A) cells. Declared co-formulants in Roundup Probio alkylpolyglycoside and nitroryl, (Table 1) are both surfactants. Declared co-formulants in Roundup Probio (Table 1) are neither classified as carcinogenic nor exert toxicity according to the material safety data sheet (MSDS).

It is not clear from this study whether the pesticide co-formulants have their own toxic effects or if they amplify the toxicity of the active ingredients. However, studies have shown that the GBH co-formulant polyethoxylated tallowamine (POE-15), is highly toxic to human cells through negatively effecting cellular respiration and membrane integrity [19]. In addition, POE-15 and the POE-15-containing GBH RangerPro have recently been demonstrated to cause cell necrosis, oxidative stress and endoplasmic reticulum stress [16]. In plants, the function of co-formulants includes increasing the solubility and stabilising the active ingredients, to facilitate cell wall penetration, and so enhance pesticidal activity [31]. Limited evidence is available to understand whether surfactants used as co-formulants can also increase the penetration of glyphosate in human cells. Nevertheless, surfactants from a Roundup formulation did not significantly influence radiolabelled glyphosate penetration over 24 h in HepG2 cells [39]. 

We previously demonstrated that the quizalofop-p-ethyl-based commercial formulation Targa Super caused a much greater decrease in cell viability than just the active ingredient after 8 days of differentiation of murine 3T3-L1 cells to adipocytes [40]. This is consistent with the results obtained here where we found that Targa Super caused a greater decrease in cell viability of both HepG2 hepatocytes (Figure 1F) and immortalised human fibroblasts (Figure 2F). In addition, an investigation of formulations of 2,4-D (2,4-D DMA) and dicamba (Banvel) found they were more toxic than their respective active ingredients in CHO cells [27,41]. Furthermore, the fluroxypyr formulation Starane 200 was demonstrated to be more toxic than fluroxypyr in HepG2 hepatocytes, HEK293 and JEG3 cells [13]. Thus, our results (Figure 1B,G and Figure 2B,G) agree with this previous study, although the formulation of fluroxypyr tested was not the same, with differences in some co-formulants, particularly the presence of naphtha. In the formulations of fluroxypyr (Hurler) and quizalofop-p-ethyl (Targa Super), declared co-formulants contain the solvent naphtha (a petroleum distillate), which has been shown to induce developmental effects in rodents [42]. Declared co-formulants of both Hurler and Targa Super (Table 1) are not classified as carcinogenic. 

We have shown that 2,4-D and to a lesser degree dicamba elicit an oxidative stress response in the ToxTracker cell assay system, and with the former also giving rise to an unfolded protein reaction [39]. In the same study we also found that glyphosate did not induce expression of oxidative stress markers. These results contrast with the findings of this investigation. On the one hand, 2,4-D did not elicit oxidative stress (Figure 4G) and dicamba only give rise to oxidative stress at relatively high concentrations (Figure 4H). On the other hand, glyphosate gave a significant oxidative stress response albeit at relatively high concentrations (Figure 4A). These differences can between the current and previous study, can be accounted for by the different outcome measures of the two assay systems: H_2_O_2_ production and reporter gene expression, with the latter likely to be more sensitive at detecting an oxidative stress response.

Given our previous investigation and thus interest in quizalofop-p-ethyl toxicity [40] and that this substance was the most potent active ingredient at stimulating oxidative stress at a lower concentration (10 μg/mL) than any of the other active ingredients tested (Figure 4F), we decided to further assess this compound’s ability to induce markers of carcinogenicity compared to its commercial formulation Targa Super using the ToxTracker assay. The results confirmed that both quizalofop-p-ethyl and Targa Super caused oxidative stress in either the absence (Figure 5A,B) or presence (Figure 5E,F) of S9 liver metabolising extract as shown by activation of the Srxn1 pathway (Sulfiredoxin 1) reflecting an Nrf2 antioxidant response, involved in the reduction of oxidized cysteines in the peroxisomes [43]. In contrast, the other biomarker for oxidative stress Blvrb (Biliverdin Reductase B) reflecting an Nrf2 independent response was not activated. This suggests that quizalofop-p-ethyl causes oxidative stress through the Nrf2 antioxidant response pathway. Quizalofop-p-ethyl also activated the unfolded protein response as shown by upregulation of Ddit3 (DNA damage-inducible transcript 3) reporter gene expression (Figure 5A,B,E,F) [40]. These results raise the concern that quizalofop-p-ethyl may act as a carcinogen and indicate the need for in vivo studies to confirm as to whether this is the case.

Although the herbicides tested in this study are the most frequently applied in the UK, it is not clear if these are also the pesticides to which humans at large are most exposed [44]. More biomonitoring studies are needed to explore this possibility, as only one study has measured herbicides in the urine of the British population [45]. In this study, which measured 186 pesticides in the urine of 130 individuals, glyphosate was the only herbicide detected with insecticides being the most frequently found to be present [45]. The high frequency of glyphosate detection can be explained by this herbicide’s use as a preharvest desiccant, which results in higher residue levels in the crop and subsequent entry into the food supply chain. In addition, exposure to herbicides is not necessarily just through the diet, as some pesticides such as dicamba are very volatile and can thus drift far from the fields they are applied, contaminating nearby populations [46]. 

The evaluation of the contribution of the co-formulants to the toxicity of formulations is a first step. However, it is important to bear in mind that human populations are exposed to mixtures of pesticides. Numerous studies stretching back many years have demonstrated that mixtures of pesticides with each at regulatory permitted doses can nevertheless be toxic [47,48,49]. 

Thus, pesticide risk assessment also needs to include mixture effects of active ingredients in combination with co-formulants present in their commercial formulations. 

Mechanisms of action other than those evaluated in this study can also contribute to carcinogenic outcomes. Endocrine disrupting pesticides such as organochlorine insecticides and the herbicide atrazine can cause carcinogenic effects by altering steroid hormone signalling in tissues, including the breast [50]. Recently, a novel mechanism which underlies the specificity of glyphosate for NHL has been demonstrated, that is upregulation of the B-cell genome mutator enzyme activation-induced cytidine deaminase [51]. Our analysis of cytotoxic effects and oxidative damage only covers a sub-set of the mechanisms by which exposure to the studied pesticides can have negative health consequences. Chlorophenoxy herbicides such as 2,4-D are toxic in mammals through different mechanisms including uncoupling of oxidative phosphorylation, and disruption of acetylcoenzyme A metabolism, resulting in a variety of symptoms such as neuromuscular paralysis [52]. Propyzamide has been found to disrupt an anti-inflammatory pathway in the digestive tract which could promote inflammatory bowel disease [53]. A recent study showed that pendimethalin altered sex steroid hormone levels, plasma vitellogenin concentration and aromatase activity in fish, suggesting it could have endocrine disrupting properties [54]. Dicamba has been demonstrated as a potential endocrine disruptor again in fish model systems, where it induced an increase in plasma vitellogenin, a change in sex hormone levels, and altered hormone-related gene expression [55]. Findings such as this should contribute to re-assessment of risks from these pesticides by regulatory agencies worldwide.

## 5. Conclusions

In conclusion, the results presented in this study show that the most frequently applied commercial herbicide formulations used in the UK and in other countries are more toxic than their corresponding active ingredients. Our data add to the increasing body of evidence, which questions the validity of health risk assessments of herbicide exposure when they are established based tests of active ingredients alone. 

## Figures and Tables

**Table 1:** Herbicide active ingredients and representative commercial formulations used in this study. The table depicts area sprayed in hectares and kilograms of active ingredients applied on arable crops grown in the UK in 2020 [34]. The declared co-formulants in the commercial formulations used in this study as reported in the material safety data sheets provided by the manufacturer is also listed. Information for quizalofop-p-ethyl and 2,4-D was not available.

Active Ingredient	Formulation	Area Spray in (Hectares)	Active Ingredients Applied (kg)	Declared Co-Formulants
Glyphosate	Roundup Probio	2,812,366	2,556,968	Alkylpolyglycoside, nitroryl, water and minor formulating ingredients
Pendimethalin	Stomp Aqua	309,809	295,480	methylenediphenyl diisocyanate, Isocyanic acid, polymethylenepolyphenylene ester (P-MDI), 4,4’-methylenediphenyl diisocyanate; diphenylmethane-4,4’-diisocyanate, Magnesium sulphate
Propyzamide	Kerb flo	228,341	105,406	Propylene glycol, 2-Naphthalenesulfonic acid, 6-hydroxy-,polymer with formaldehyde and methylphenol, sodium salt
Metazachlor	Sultan 50C	51,656	30,958	-
Fluroxypyr	Hurler	675,157	83,041	BUTANOL-norm, Hydrocarbons, C9, aromatics, Organic sulfonate–proprietary, Solvent naphtha (petroleum), light aromatic
Dicamba	Hunter	76,083	39,591	-
Quizalofop-p-ethyl	Targa Super	N/A	N/A	Calcium dodecylbenzene sulphonate, Ethoxylated lauryl alcohol C12, Solvent naphtha (petroleum), super heavy aromatic (<1% naphthalene), solvent naphtha (petroleum), heavy aromatic (<1% naphthalene)
2,4-Dichlorophenoxyacetic acid (2,4-D)	Anti-liserons	N/A	N/A	-

## Data Availability

Not applicable.
